# A mobile-phone based high-resolution microendoscope to image cervical precancer

**DOI:** 10.1371/journal.pone.0211045

**Published:** 2019-02-06

**Authors:** Benjamin D. Grant, Timothy Quang, Júlio César Possati-Resende, Cristovam Scapulatempo-Neto, Graziela de Macedo Matsushita, Edmundo Carvalho Mauad, Mark H. Stoler, Philip E. Castle, José Humberto Tavares Guerreiro Fregnani, Kathleen M. Schmeler, Rebecca Richards-Kortum

**Affiliations:** 1 Department of Bioengineering, Rice University, Houston, Texas, United States of America; 2 Department of Cancer Prevention, Pio XII Foundation, Barretos Cancer Hospital, Barretos, Brazil; 3 Molecular Oncology Research Center, Pio XII Foundation, Barretos Cancer Hospital, Barretos, Brazil; 4 Department of Pathology, Pio XII Foundation, Barretos Cancer Hospital, Barretos, Brazil; 5 Department of Pathology, University of Virginia School of Medicine, Charlottesville, VA, United States of America; 6 Global Coalition against Cervical Cancer, Arlington, VA, United States of America; 7 Department of Epidemiology and Population Health, Albert Einstein College of Medicine, Bronx, NY, United States of America; 8 Center for the Researcher Support, Pio XII Foundation, Barretos Cancer Hospital, Barretos, Brazil; 9 Department of Gynecologic Oncology and Reproductive Medicine, The University of Texas MD Anderson Cancer Center, Houston, TX, United States of America; University of South Carolina, UNITED STATES

## Abstract

Nearly 90% of cervical cancer cases and deaths occur in low- and middle-income countries that lack comprehensive national HPV immunization and cervical cancer screening programs. In these settings, it is difficult to implement screening programs due to a lack of infrastructure and shortage of trained personnel. Screening programs based on visual inspection with acetic acid (VIA) have been successfully implemented in some low-resource settings. However, VIA has poor specificity and up to 90% of patients receiving treatment based on a positive VIA exam are over-treated. A number of studies have suggested that high-resolution cervical imaging to visualize nuclear morphology *in vivo* can improve specificity by better distinguishing precancerous and benign lesions. To enable high-resolution imaging in low-resource settings, we developed a portable, low-cost, high-resolution microendoscope that uses a mobile phone to detect and display images of cervical epithelium *in vivo* with subcellular resolution. The device was fabricated for less than $2,000 using commercially available optical components including filters, an LED and triplet lenses assembled in a 3D-printed opto-mechanical mount. We show that the mobile high-resolution microendoscope achieves similar resolution and signal-to-background ratio as previously reported high-resolution microendoscope systems using traditional cameras and computers to detect and display images. Finally, we demonstrate the ability of the mobile high-resolution microendoscope to image normal and precancerous squamous epithelium of the cervix *in vivo* in a gynecological referral clinic in Barretos, Brazil.

## Introduction

Cervical cancer remains a major global health concern with an estimated 527,600 new cases and 265,700 deaths attributed to cervical cancer in 2012. Nearly 90% of all cervical cancer deaths occurred in developing countries [1]. In countries with the financial and human resources to support national screening [2], the incidence and mortality of cervical cancer has dropped drastically. Screening prevents cervical cancer by allowing for the detection and treatment of precursor lesions [2]. Although an effective HPV vaccine has been developed[3–5], implementation in low resource implementation is limited largely due to the high cost of the vaccine [6]. Furthermore, the vaccine is not effective for women who are already infected with HPV. Screening will continue to remain important for decades for those that did not receive the vaccine prior to infection and for those infected with oncogenic HPV types the vaccine does not protect against [7]. Unfortunately, in low- to middle-income countries (LMICs) screening programs are difficult to implement due to a lack of human and financial resources and inadequate infrastructure [8].

Alternative, low-cost screening techniques appropriate for LMICs have been developed to decrease the global burden of cervical cancer. Visual inspection with acetic acid (VIA) and visual inspection with Lugol’s iodine (VILI) are two alternative screening techniques [8]. In VIA, a healthcare provider applies dilute acetic acid directly to the cervix and examines the cervix for signs of acetowhitening, a localized whitening of the epithelium associated with abnormal underlying tissue [9]. In VILI, dilute Lugol’s iodine is applied topically to the cervical epithelium, staining normal glycogen-rich squamous epithelium preferentially, leaving glycogen-depleted proliferative cancerous and precancerous lesions unstained [10]. More recently, human papillomavirus (HPV) DNA testing has been explored for cervical cancer screening. HPV DNA testing is highly sensitive [11] because virtually every case of cervical cancer is caused by a persistent high-risk HPV infection [12]. However, VIA, VILI and HPV DNA testing are all limited by high false-positive rates and low positive-predictive values [10,13–15], leading to substantial overtreatment when used as part of a see-and-treat strategy.

Various optical imaging techniques have also been explored to allow for identification of cervical precancer *in vivo* [16]. One such technique is the high-resolution microendoscopy [17–19]. The high-resolution microendoscope (HRME) is a fiber bundle-based fluorescence microscope capable of imaging cell nuclei *in vivo*. Images are acquired following topical application of the fluorescent topical contrast agent proflavine, which selectively stains cell nuclei. Images obtained with the HRME device can be used to quantify nuclear features associated with neoplastic progression, such as nuclear-to-cytoplasmic ratio and nuclear eccentricity [20,21]. In pilot studies in China [20], Botswana [21] and Brazil[22], the HRME identified cervical pre-cancer with high sensitivity and specificity. However, the cost ($5,000) and size (28cm x 23cm x 6.5cm) of the device preclude its use in some low-resource settings. The cost and size of the standard HRME are driven by three main components: a scientific-grade camera, a laptop computer, and off-the-shelf optomechanics [18,19]. Recent advances in mobile phone technology, including high processor speeds and compact digital cameras, provide a unique opportunity to simultaneously reduce both the size and cost of the HRME. Due to the ubiquity of mobile phones and their increasingly sophisticated cameras, other research groups have designed mobile phone-based microscopes [23,24]. Here, we report a low-cost, highly portable mobile HRME (mHRME) utilizing a mobile phone and custom 3D-printed parts in lieu of a scientific-grade camera, laptop and commercial optomechanics.

## Materials and methods

### Optical design

The mHRME is a fiber-based fluorescence microscope, similar in operation to that described by Muldoon *et al*. [18]. The mHRME consists of four main components: a light emitting diode (LED) to illuminate tissue, a one millimeter outer-diameter coherent fiber bundle to deliver illumination light and collect resulting fluorescence, a mobile phone to record and display the fluorescence image, and optical lenses and filters to direct light through the system. The device was designed with a target magnification of 1.0 to ensure that the entire field-of-view (FOV) of the fiber could be captured within the dimension of the complementary metal-oxide-semiconductor (CMOS) sensor of the cell phone. To minimize distortion, Steinheil lenses were selected for all of the imaging optics because they are optimized for 1:1 conjugate ratios. To create a compact, portable device, all lenses had a maximum diameter of no more than 12.5 mm. ZEMAX was used to aid in commercial lens selection and positioning.

**Fig 1:**
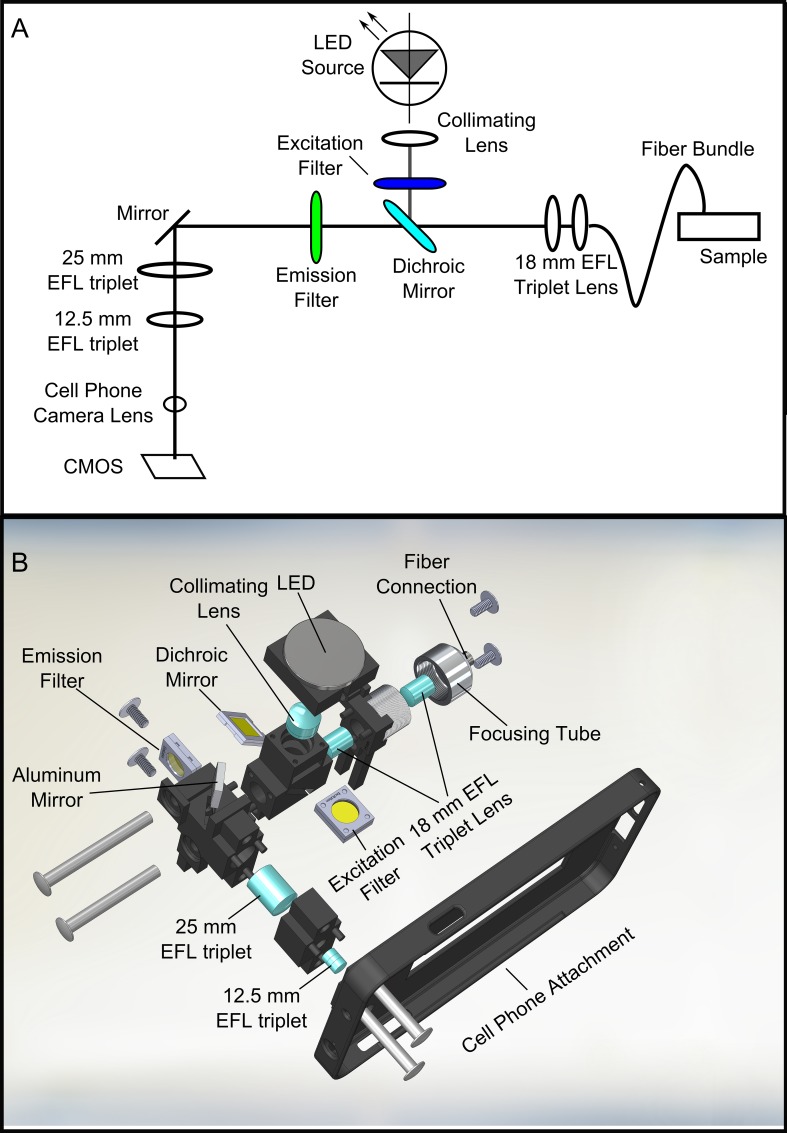
Schematic of mHRME. **(A)** Diagram of optical layout of the system. **(B)** Solidworks illustration depicting all components of the system.

An overview of the mHRME optical pathway is shown in **Fig 1A**. An LED (LED Engin, San Jose, California, LZ1-10DB00) provides excitation light centered at 460 nm. The LED is powered in one of two ways. For cell phones that offer USB on-the-go support, the LED is powered through a USB cable connected to the phone. For phones lacking this capability, an external USB battery pack (e.g., Anker 10,000 mAh battery pack) is used. The current is regulated by a 1000 mAh LED driver (LED Supply, Randolf, VT, 03023-D-E-1000P). The LED is collimated through an achromatic doublet (Edmund Optics, Barrington, NJ, 65–550) before passing through a 20 nm band-pass excitation filter centered at 448 nm (Edmund Optics, Barrington, NJ, 86–975). The excitation light is then reflected towards the fiber bundle via a dichroic mirror with a 482 nm cut-on wavelength (Edmund Optics, Barrington, NJ, 86–317). After reflecting off the dichroic mirror, the light is coupled to a fiber bundle (Fujikura, Tokyo, Japan, FIGH-30-850N) by a pair of 18 mm effective focal length (EFL) triplet lenses (Edmund Optics, Barrington, NJ, 45–399). The fiber bundle is composed of 30,000 fibers with 4 μm spacing between each fiber. The dimensions of the fiber bundle determine the field-of-view of the system.

The system is designed to be used with the fiber bundle placed in direct contact with proflavine-stained epithelial tissue. Fluorescence light emitted from the tissue following excitation is collected through the same fiber. After passing through the fiber, emission light is directed through the pair of triplet lenses prior to transmitting through the dichroic mirror. Next, the emitted light is filtered by a 515 nm long-pass color glass emission filter (Schott OG-515). The remaining signal is redirected with an aluminum mirror (Thorlabs ME05S-G01) through a 25mm EFL triplet lens (47–675 Thorlabs) and is coupled to the lens of the cell phone through a 12.5 mm EFL triplet.

Based on the optimal lens positions identified in ZEMAX, a cell-phone attachment and optomechanic holder was printed with a 3D printer (3D Systems, Rock Hill, South Carolina). The 3D-printed structure consists of individual pieces that snap together as shown in **Fig 1B**. The pieces contain cutouts corresponding to the exact size of the lenses, ensuring that the lenses are positioned accurately and precisely. After assembly, four threaded aluminum rods are placed in through-holes to ensure stability during use. The optical system connects directly to the phone, thereby ensuring alignment of the optics with the mobile phone camera. We tested the mHRME using two phones: the Galaxy Note 3 (Samsung, Seoul, South Korea) which features a 13 megapixel camera and 2.3 GHz processor and the One X+ (HTC, New Taipei, Taiwan), an older, midlevel international smartphone with 8 megapixel resolution.

### Imaging testing

#### In vitro imaging

We first determined the resolution of the system using a standard Air Force Resolution Target at Rice University. The fiber probe was placed on a translational stage and put in direct contact with an Air Force Resolution Target. ImageJ (National Institutes of Health, Bethesda, Maryland) was used to determine the smallest line pairs that were distinguishable in the acquired images. Next, to validate the ability of the mHRME to image cell nuclei and to assess the signal-to-background ratio (SBR) we evaluated its performance in cultured HeLa cells (ATCC, Manassas, VA). HeLa cells were cultured in Dulbecco’s Modified Eagle’s Medium (DMEM) in a 150 mm Petri dish until approximately 50% confluent. The media was aspirated and the cells were washed once with phosphate buffered saline (PBS). After aspirating the PBS, 1 mL of 0.01% proflavine (Sigma-Aldrich, St. Louis, MO) in PBS was applied to cover the cells. After one minute, the proflavine was removed and cells were washed twice with PBS. The cells remained in PBS solution and were imaged with the mHRME system directly in the Petri dish; the tip of the fiber was placed in direct contact with the cells.

#### In vivo imaging

After successfully imaging cultured cells, we proceeded to test the mHRME *in vivo*.

Patients with abnormal Pap smears referred to Barretos Cancer Hospital (Barretos, Brazil) for colposcopy were enrolled in a pilot study to evaluate the efficacy of the standard HRME in discriminating between low-grade and high-grade lesions [25]. Two patients in this study were also imaged with our mHRME demonstrated its ability to achieve sub-cellular resolution in vivo. The National Committee for Ethics in Research (CONEP) of Brazil, the Research Ethics Committees of Barretos Cancer Hospital and the Institutional Review Boards from MD Anderson Cancer Center and Rice University approved this study. Written informed consent was obtained for all participants.

During routine colposcopy, the colposcopist first evaluated the cervix using 5% acetic acid under white-light conditions. Then, the colposcopist applied 0.01% proflavine solution to the cervix using a spray bottle and cotton swab. After the application of proflavine, Lugols’ iodine was applied as part of the standard of care for colposcopy at Barretos Cancer Hospital. Proflavine was then applied again to enhance signal strength. Next, the tip of the mHRME probe was placed in gentle contact with an area of the cervix that appeared normal by colposcopy. Images were captured using the android app Camera FV-5 (FGAE studios, Stuttgart, Germany). After acquiring images of normal squamous epithelium, mHRME images were collected from colposcopically abnormal areas.

## Results

### System characterization

**Fig 2:**
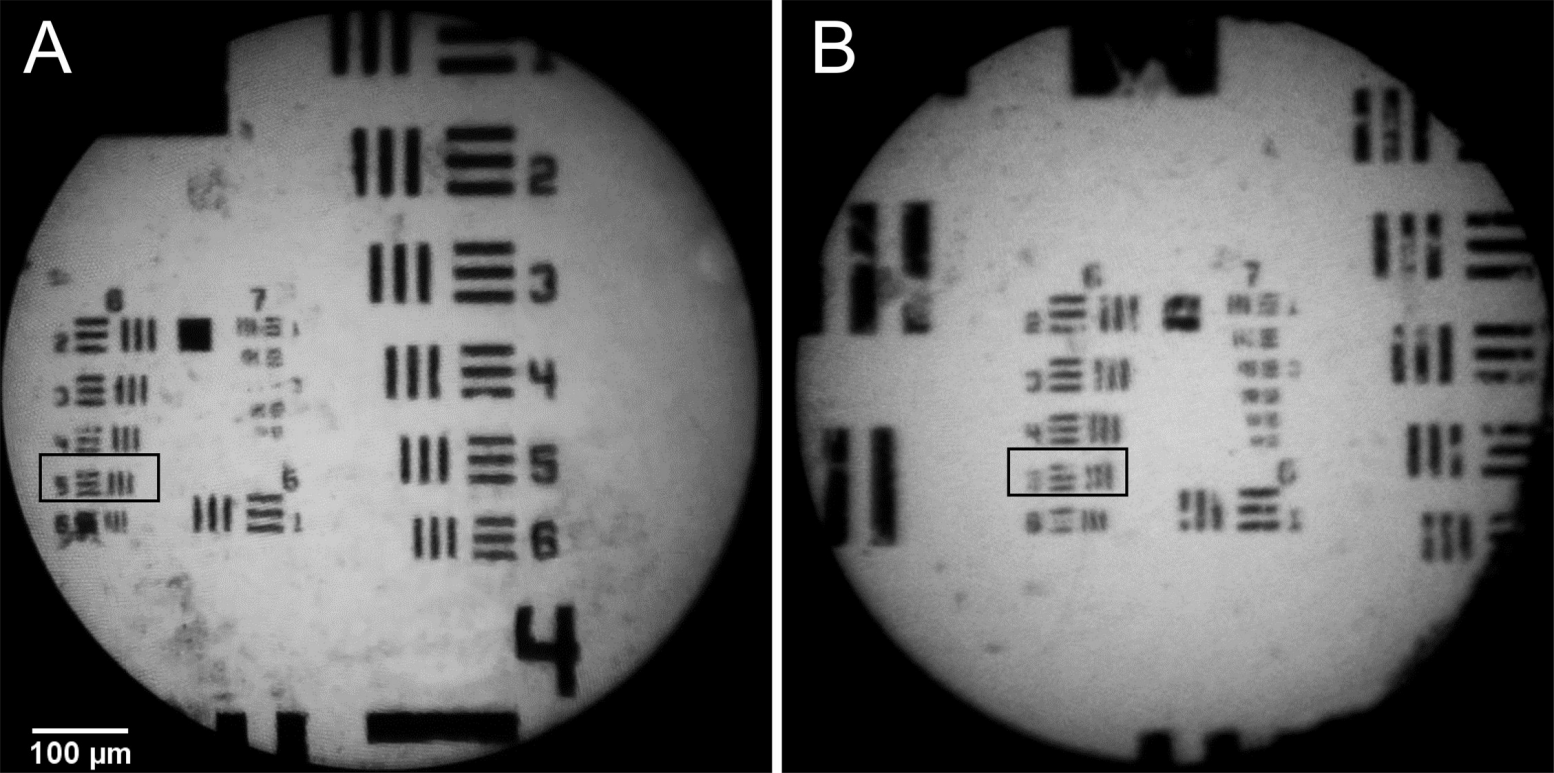
**Air-Force Resolution Target image taken with the mHRME system using (A)** the Galaxy Note 3 and **(B)** The HTC One X+ The smallest target the system can resolve is group 6, element 5 (boxed) corresponding to a resolution of 4.9 μm.

The key properties of the system are outlined in **Table 1**. The device, excluding the mobile phone, weighs 170 g and is 15 cm x 7 cm x 8 cm using the Galaxy Note 3. The optical power output at the distal end of the fiber is 0.8 mW/cm^2^ and the field-of-view is 750 μm. The system is capable of resolving element five of group six, as shown in **Fig 2** using both the Galaxy Note 3 and HTC One X+; this element corresponds to a line-width of 4.9 μm.

**Table 1:** Key system parameters for the standard HRME[26] and mHRME using the Galaxy Note 3 and HTC One X+.

	Standard HRME	mHRME–Galaxy Note 3	mHMRE–HTC One X+
Image Sensor Size (diagonal)	11 mm	5.87 mm	5.68 mm
Spatial Resolution	4.4 μm	4.9 μm	4.9 μm
Fiber Bundle Size	30,000 fiber cores	30,000 fiber cores	30,000 fiber cores
Pixels per fiber	27 pixels	20.7 pixels	9.8 pixels
Field-of-View	750 μm	750 μm	750 μm
Weight*	2300 g	170 g	170 g
Dimensions	20 cm x 25 cm x 6.4 cm	15 cm x 7 cm x 8 cm	13 cm x 7 cm x 8 cm
Prototype Cost	$5,000	$1,650	$1,350

Both mHRMEs are significantly smaller, lighter and less expensive than the standard HRME system.

*Weight excludes the weight of the laptop or mobile phone

### Cultured cell imaging

**Fig 3:**
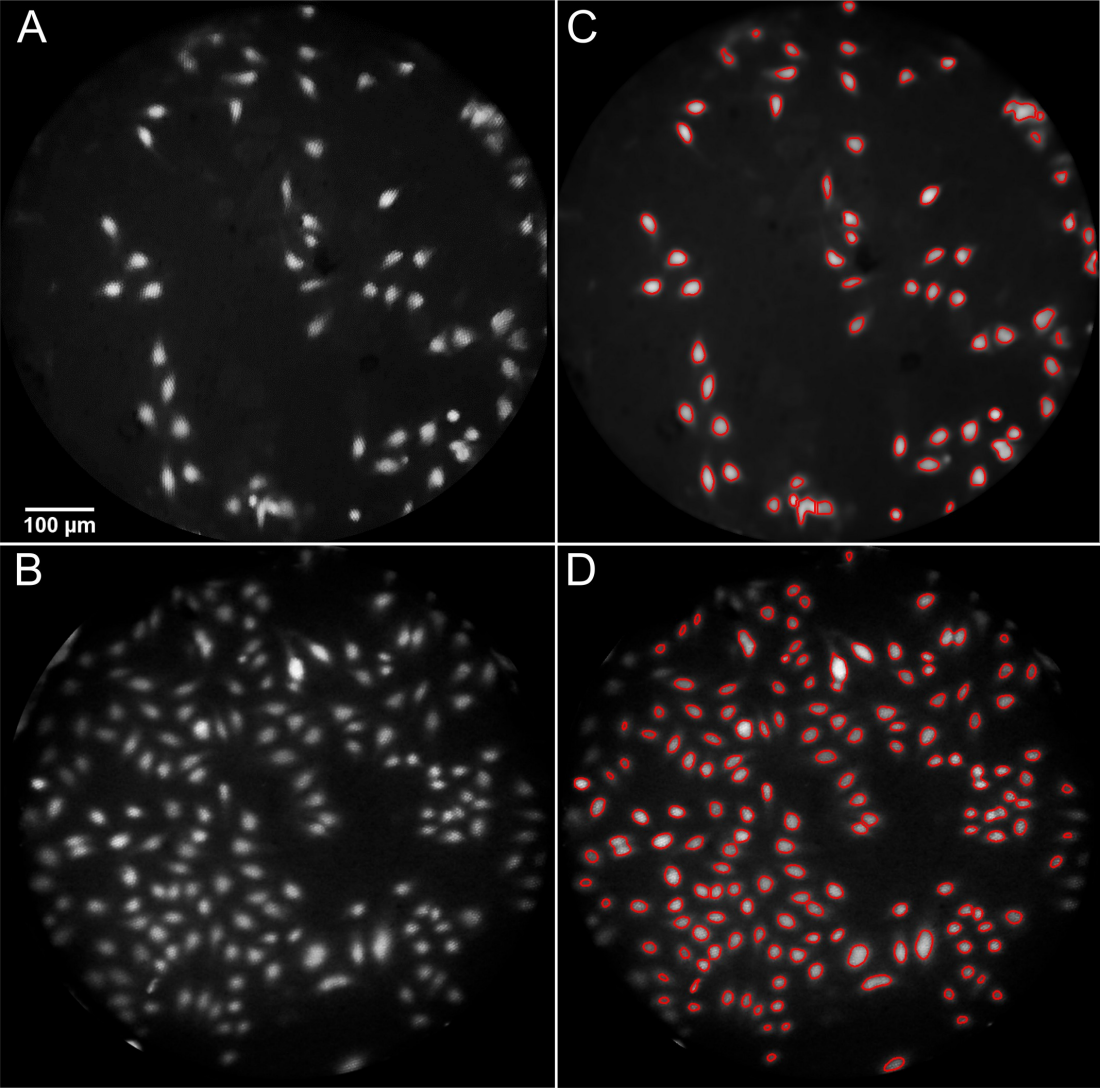
HeLa cell imaging. Raw images of cultured HeLa cells taken with the **(A)** Galaxy Note 3 and **(B)** HTC One X+ mobile high resolution microendoscopes. Nuclei from **(A)** and **(B)** are automatically selected from the background using a matlab scripts and outlined in (**C)** and **(D),** respectively.

Images of proflavine-stained HeLa cells taken with the Galaxy Note 3 and HTC One X+ are shown in **Fig 3.** The figure shows the green channel from an otherwise unprocessed mHRME image. In order to evaluate the signal-to-background ratio, the cells were segmented automatically from the background using a custom MATLAB script. First, a top-hat filter was applied to reduce the background and correct any uneven illumination. Next, a threshold was determined using Otsu’s method [27] to separate nuclei from the background of the image. The resulting segmentation is shown in **Fig 3**. The signal-to-background ratio was calculated by dividing the mean intensity of pixels within the segmented cell nuclei by the mean intensity of pixels within the fiber bundle not classified as nuclei. The resulting signal-to-background ratio is 5.3 for the Galaxy Note 3 system and 4.4 for the HTC One X+ system, comparing favorably to that obtained with previous standard HRME systems [17,18].

### In vivo imaging

**Fig 4:**
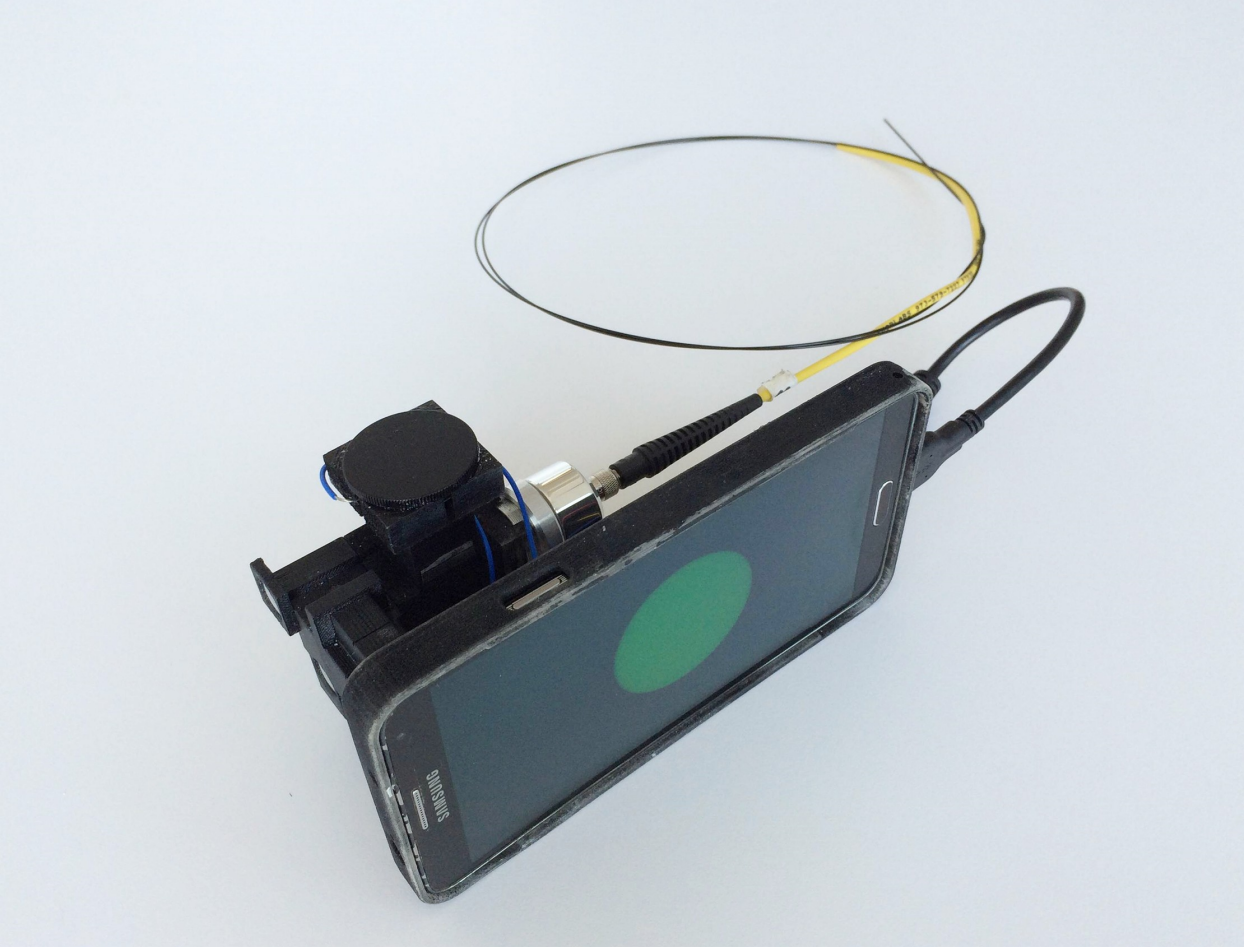
Photo of the assembled prototype.

**Fig 5:**
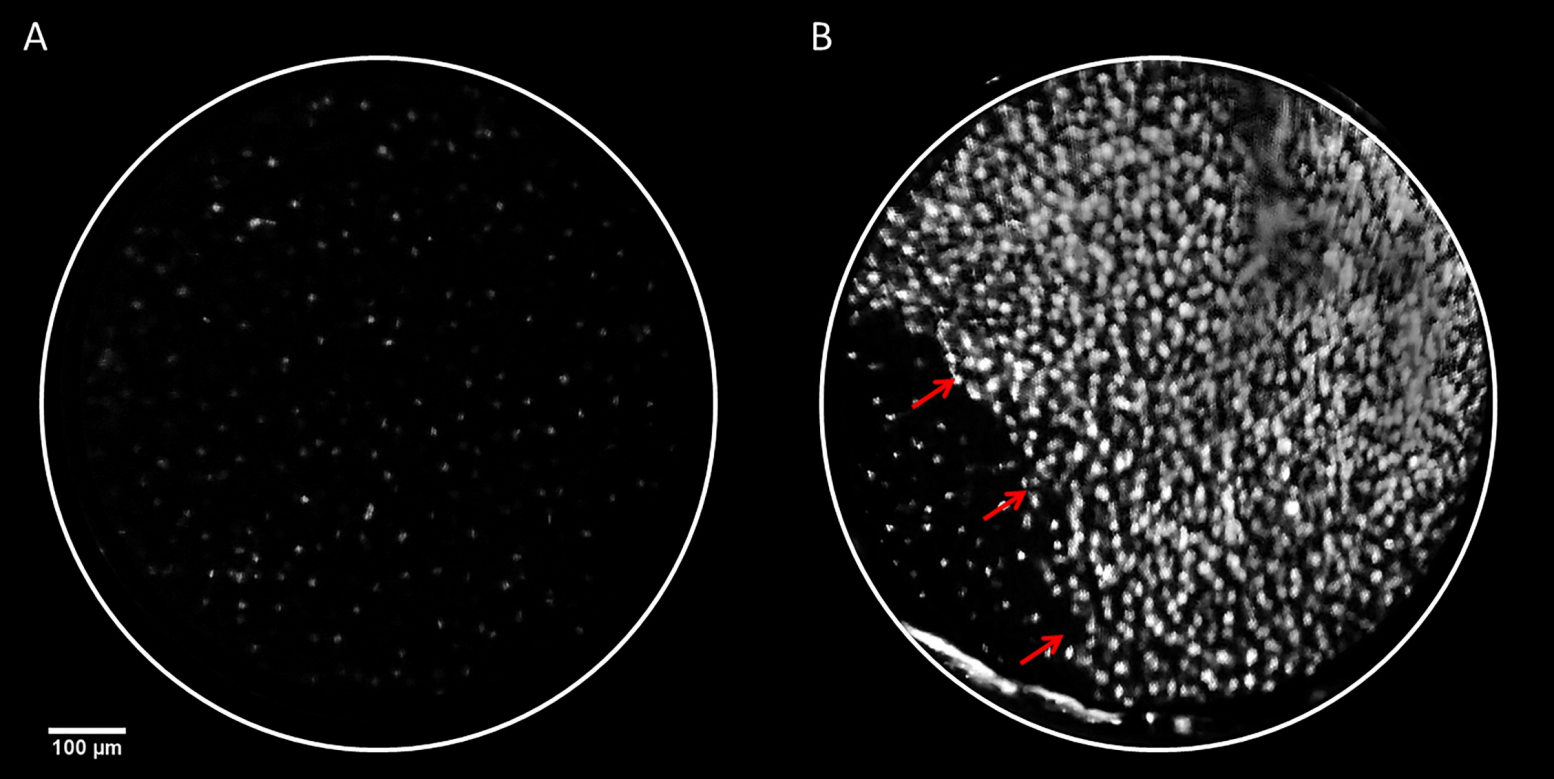
*In vivo* imaging of the cervix with the mHRME of squamous cell epithelium with morphology consistent with (A) normal squamous epithelium and (B) high grade dysplasia. The white circle indicates the boundary of the fiber bundle. The red arrows in **(B)** indicate the junction between the lesion and surrounding normal tissue.

The mHRME was tested by colposcopists at Barretos Cancer Hospital to determine its ability to image squamous epithelium *in vivo*. **Fig 4** shows the completed prototype with the Galaxy Note 3 mobile phone to illustrate the device’s overall size and design. **Fig 5** provides representative *in vivo* mHRME images obtained from colposcopically normal and colposcopically abnormal squamous epithelium. The mHRME image of colposcopically normal tissue shows round, well-spaced and small nuclei, consistent with normal squamous epithelium. In contrast, the majority of the mHRME image of colposcopically abnormal tissue shows large, pleomorphic, crowded nuclei, consistent with high-grade dysplasia[22,28]. This image was taken at the edge of a visible lesion; the junction between abnormal and normal epithelium is evident in the bottom left hand region of **Fig 5B** and is marked with arrows. Both mHRME images in **Fig 5** have been post-processed identically by selecting only the green channel, applying a top-hat filter, and enhancing the contrast.

## Discussion

We developed a mobile phone high-resolution microendoscope capable of imaging epithelial cells *in vivo*. At 4.9 μm, the lateral resolution is slightly inferior to that of the standard HRME system (4.4 μm[26]), likely due to the smaller, lower quality camera sensor. Additionally, we are not using the full sensor of the cell phone camera to ensure that the entire field-of-view is visible across phones with different sensor sizes. However, the resolution of the system is sufficient to acquire sub-cellular resolution images of cervical tissue *in vivo*. Using a mobile phone eliminates the need for a scientific-grade camera and laptop, thereby significantly reducing both size and cost and increasing portability. Although image processing is currently done off the phone, in the future this post-processing could be achieved in near real-time with a custom android camera application. The battery-powered system can be used in healthcare settings without wall electricity. Using the Galaxy Note 3’s built-in battery provides two hours of imaging, while the economical (~$30) Anker external USB battery pack allows for ten hours of imaging.

The system cost could be reduced substantially if produced in large quantities. First, 3D-printed components could be made by injection molding at a fraction of the price. Second, the optical lenses and LEDs drop markedly in price when purchased in bulk. With large-scale production the price of the mHRME would be under $500, not including cost of the cell phone. Phones with cameras equivalent or greater specifications than the 2012 HTC one X+’s should be sufficient to obtain images with sub-cellular resolution. Due to these relatively low requirements, old smart phones could be recycled solely for use in a mHRME significantly reduced cost. Because the device does not have any associated consumables, we believe this price target is reasonable even for use in low-resource settings.

The device is also designed to be flexible to allow for different applications. The LED, filters and dichroic mirror can be changed in order to utilize other fluorescent dyes without any additional system modifications. The field-of-view can be changed depending on the application by using different fiber bundles. For applications in which a much larger field-of-view is desirable, mosaicking approaches can be implemented [29]. Finally, the optics of the system are designed to interface with a variety of mobile phones. The magnification was chosen such that the CMOS sensor is underfilled. This ensures that if other mobile phones were selected with smaller sensor sizes, the image of the full fiber bundle would still fit on the sensor. Light from the mHRME is collimated before it is directed to the cell phone camera and integrated optics. A cell phone with a camera of at least 8 megapixels that can be set to infinity or landscape mode is sufficient to acquire in-focus images with subcellular resolution.

In conclusion, we have demonstrated a mobile phone-based microscope capable of achieving sub-cellular resolution images of cervical epithelium *in vivo*. While initial results are promising, further studies are necessary to validate that the device can identify cervical precancer with high sensitivity and specificity to evaluate its diagnostic performance. Advances in mobile phone technology and rapid prototyping techniques present an exciting opportunity to develop new diagnostic imaging techniques tailored for use in low-resource settings. We believe this device may have particular utility in countries in which traditional cervical cancer screening techniques are difficult to implement.
